# The use of platelet rich plasma in the treatment of degenerative joint disease in cats: an exploratory case series

**DOI:** 10.3389/fvets.2024.1394055

**Published:** 2024-05-28

**Authors:** Janice Huntingford, Andrea Looney, James Johnson, Lisa Miller

**Affiliations:** ^1^Essex Animal Hospital, Essex, ON, Canada; ^2^Central Hospital for Veterinary Medicine, North Haven, CT, United States; ^3^Companion Animal Health, New Castle, DE, United States

**Keywords:** platelet rich plasma (PRP), feline, degenerative joint disease (DJD), osteoarthritis, regenerative medicine

## Abstract

**Objective:**

To evaluate the effectiveness of intra-articular autologous Platelet Rich Plasma (PRP) in managing Degenerative Joint Disease (DJD) in cats.

**Design:**

Prospective pilot clinical trial.

**Methods:**

Six domestic cats with clinically and radiographically diagnosed DJD received intra-articular injections of autologous PRP. Clinical assessments pre and post intra-articular injections were conducted using the Feline Musculoskeletal Pain Index (FMPI, owner assessed) and Visual Analog Scale (VAS, clinician assessed) at baseline, Day 14, Day 28, and Day 42–45.

**Results:**

Significant improvements were noted in both FMPI and VAS scores at the end of the study period, indicating enhanced joint function and reduced pain.

**Conclusion and clinical relevance:**

The study suggests the potential of PRP therapy as a safe and effective treatment for feline DJD, warranting further research with larger cohorts and longer follow-up to establish comprehensive treatment guidelines.

## Introduction

Degenerative Joint Disease (DJD) in cats is a prevalent condition, significantly affecting the quality of life of aging feline populations (1). This chronic disease often presents with subtle clinical signs, making diagnosis challenging and frequently leading to underdiagnosis. Current treatments primarily focus on pain management through medications and physical rehabilitation, but these approaches have limitations, including variable efficacy and potential side effects (2, 3). The need for more effective and safe therapeutic options is clear, prompting exploration into alternative minimally invasive treatments such as intra-articular Platelet Rich Plasma (PRP) therapy.

Platelet Rich Plasma therapy, an innovative approach in regenerative medicine, harnesses the healing powers of platelets and growth factors present in blood (4, 5). Historically, PRP has been utilized in human medicine for various orthopedic conditions, including osteoarthritis (OA) and connective tissue injuries, due to its potential to enhance tissue repair and regeneration (6–8). The therapy involves concentrating platelets from the patient’s own blood and reinjecting them into affected areas, aiming to stimulate a natural healing response (9). PRP’s mechanism of action, leveraging growth factors, and altering immune response to modulate inflammation and promote tissue healing (10), positions it as a promising candidate for addressing orthopedic conditions in veterinary medicine.

In the field of veterinary medicine, PRP therapy has gained attention as a promising treatment for various orthopedic conditions in animals (11–16). Studies in canine and equine models have demonstrated encouraging outcomes, particularly in the treatment of OA and tendon injuries (17, 18). These studies suggest that PRP can enhance joint function and reduce pain, thereby improving the quality of life in these animals. Unfortunately, no studies have investigated the effect of PRP as a treatment for feline DJD/OA.

While the benefits of PRP in veterinary medicine have been documented, it is important to differentiate between types of PRP products and commercially available preparation systems. A recent review outlined the mechanism of action, different PRP types, and some of the commercial systems available for clinical therapy isolation and administration (19). As outlined in that work and others (20–22), clinical efficacy is dependent on preparation/isolation methods and the equipment used therein. Thus, studies investigating the clinical efficacy of this therapy must consider preparation effects.

The rationale for this study arises from the need to explore effective and safe treatments for DJD in cats, a condition that remains challenging to manage with current therapies (1). Given the promising results of PRP in other species, this study aims to investigate its potential efficacy and safety in feline DJD. The primary objective is to assess the impact of PRP on joint function and pain relief in cats suffering from DJD, with the hypothesis that PRP treatment will lead to significant clinical improvements, thus offering a valuable alternative to current treatment strategies.

## Materials and methods

This study was approved by the Ethics Committee and the clinical trial committee at both study sites: Essex Animal Hospital (Essex, ON, Canada) and Central Hospital for Veterinary Medicine (North Haven, CT, United States), respectively. Written owner consent was granted in each case following verbal discussion of the study. Data are reported in compliance with pertinent CONSORT guidelines.

### Study design

This was a prospective pilot clinical trial. Outcome measures included changes in owner ratings of various daily functions and activities, as well as changes in clinician pain scoring before and after having been treated with intra articular platelet rich plasma.

### Animals

Potential study subjects were identified from clinic records or were referred by primary care veterinarians that had seen advertisements for the clinical trial. Animals enrolled in the study were all client owned animals with naturally occurring chronic musculoskeletal disease.

### Screening

Patients were eligible to participate in the study if they were greater than 1 year of age and weighed more than 2.27 kg (5.0 lb). had one or more symptoms of chronic pain related to DJD/OA noted by the pet-owner, evidence of pain during manipulation of at least two joints during veterinary orthopedic evaluation and radiographic evidence of degenerative joint disease in at least one of the painful joints.

Exclusion criteria for all patients included the presence of suspected or diagnosed infectious diseases, symptomatic cardiac disease, immune-mediated disease, neoplasia, active urinary tract infection, hyperthyroidism, chronic kidney disease, and diabetes mellitus. These conditions were ruled out by review of medical records, owner provided history, physical examination, complete blood count (CBC), serum biochemistry panels, and urinalysis. Patients were also excluded if baseline CBC indicated thrombocytopenia.

Pet owners were asked to keep the patients in the same household, on previously prescribed foods, supplements and daily medications without changes, throughout the entirety of the study period (no moving/relocation) and were not eligible if they had any anticipated significant disturbances to normal daily routine (new baby in household, new pets in household, etc.) during study period.

Orthopedic evaluations were performed by a veterinarian at each study site (Central Hospital for Veterinary Medicine and Essex Animal Hospital). Both veterinarians performing evaluation were board certified diplomates of the American College of Veterinary Sports Medicine and Rehabilitation. Physical exam was performed on each patient on an examination table and pertinent to the study, included joint palpation through range of motion, observation of gait, neurologic exam, and strength exam. Patients that met eligibility criteria for owner-noted activity impairment or perceived pain and pain on orthopedic evaluation were sedated using a standard protocol and orthogonal radiographs were taken of identified problematic joints and/or spinal segment. Radiographs were reviewed for the presence of degenerative joint disease by the attending veterinarian.

### Study timeline

Cats were screened on Day 0 with pet owner-interview, physical, orthopedic examinations, radiographs, and lab work (CBC, serum biochemistry panel, urinalysis, and T4) unless lab work and radiographs had been completed previously in the past 6 months. Enrolled patients received the outlined treatment, and completed outcome measure questionnaires on Days 0, 14, 28, and 42–45 as specified.

### Platelet rich plasma administration

Autologous PRP was processed using the CRT system and 30 mL Pure PRP kits (Companion Animal Health). A volume of 12.5 mL of whole blood was collected from the jugular vein into a 30 mL syringe via 22 g butterfly catheter primed with Anticoagulant Citrate Dextrose Solution (ACDA) 2.5 m within the syringe. The combined whole blood and ACDA was then processed in the concentrating devices according to manufacturer instructions. Each patient was sedated for intra-articular (IA) injection of the final PRP solution. Any joint area(s) identified for treatment were clipped and aseptically prepared. The confirmation of correct needle placement was obtained by collecting synovial fluid. If required, ultrasound guidance was available to confirm correct needle placement. As much synovial fluid as possible was withdrawn, and PRP was administered. For the hip, stifle, or shoulder joint(s) a volume of 0.5 mL PRP was injected. For the elbow joint(s), a volume of 0.25 mL PRP was injected.

### Outcome measurement

For each cat, measured outcomes included changes in owner assessment using a clinical metrology instrument questionnaire (FMPI version 9, NC state) as described previously (23) and clinician assessment of lameness and pain utilizing VAS score (assessed during comprehensive patient examination performed during each visit outlined in Methods/Screening section). Briefly, owners completed the FMPI questionnaire and clinicians scored the cats using VAS on day 0 for baseline assessments and subsequent assessments occurred on day 14 (week 2), day 28 (week 4), and day 42–45 (week 6). Owners were counseled to recognize signs of pain in their cats and instructed on use of the questionnaire prior to each assessment. This questionnaire has been used in previous studies of cats with DJD/OA (24) and its development has been described (25). The FMPI queries owners on their cat’s ability to perform each of 17 activities (rated on a Likert scale from “Normal” = 4 to “Not at all [able to]” = 0) with an option to select “Do not know or Not Applicable.” Within questions 18 and 19 of the FMPI, pet owners were requested to assess their patient’s pain level for the preceding 2 weeks and for the present day, respectively, utilizing a standardized 100 mm visual analog scale (VAS). Visual analog scale scores were computed by measuring the distance in millimeters from the starting point (zero) to the mark made by the owner, where 100 represented worst pain possible. The scores were then divided by 25 to align the scales across the entire instrument. The potential score range encompassed values from 0 to 68 for items 1–17 and extended to 0–78 for the complete FMPI assessment.

Information derived from the FMPI was summarized according to categories, including raw scores (total FMPI points for Q1–17 and Q1–19) and percent possible scores (%poss). Percent possible scores were employed to account for situations where certain owners were unable to respond to all items, such as owners who did not have stairs and indicated a “Not Applicable” response for stair-related items. The calculations for percent possible scores for items 1–17 and 1–19 were determined using the following equations, respectively (23):


$$\begin{array}{l} \mathrm{FMPI}\%\mathrm{poss}\;\mathrm{Score}\;Q1-17\\ =\left(\Sigma Q1-17\;\mathrm{scores}\right)÷\left(\mathrm{number}\;\mathrm{of}\;\mathrm{questions}\;\mathrm{answered}\times 4\right) \end{array}$$

$$\begin{array}{l} \mathrm{FMPI}\%\mathrm{poss}\;\mathrm{Score}\;Q1-19=\left(\left(\Sigma Q1-17\;\mathrm{scores}\right)+\left(Q18÷25+Q19÷25\right)\right)\\ ÷\left(\mathrm{number}\;\mathrm{of}\;\mathrm{questions}\;\mathrm{answered}\times 4\right) \end{array}$$

Clinician VAS pain scoring was performed on the same scale as described above and results were converted by dividing by 25 to align the scales for the entire instrument. Q18–19 FMPI scoring were only completed at the 0- and 6-week timepoints, whereas the Q1–17 FMPI and clinician VAS scoring was completed at the 0-, 2-, 4-, and 6-week timepoints. Adverse events were recorded throughout the study.

### Statistical analysis

FMPI Q1–17, FMPI%poss Q1–17, and VAS scores were compared using a mixed effects (paired) analysis with Dunnett multiple comparisons test to compare the 2-, 4-, and 6-week outcomes to the 0-week baseline (α = 0.05). FMPI Q1–19 and FMPI%poss Q1–19 scores were compared using a two-tailed paired *t*-test analysis compare the 6-week outcomes to the 0-week baseline.

## Results

### Patient enrollment/demographics

Recruitment and patient treatment occurred between July 2021 and April 2022. A total of eight cats were screened and six were deemed eligible for the study. Two patients were excluded due to the presence of baseline lab work abnormalities suggesting systemic disease. Of six patients enrolled, the patient age was 9.38 ± 4.37 years and mean body weight was 6.30 ± 1.68 kg. The most common joint injected was the elbow (*n* = 5) with four of six cats requiring injection of the same joint bilaterally.

### FMPI scoring results

**Figure 1:**
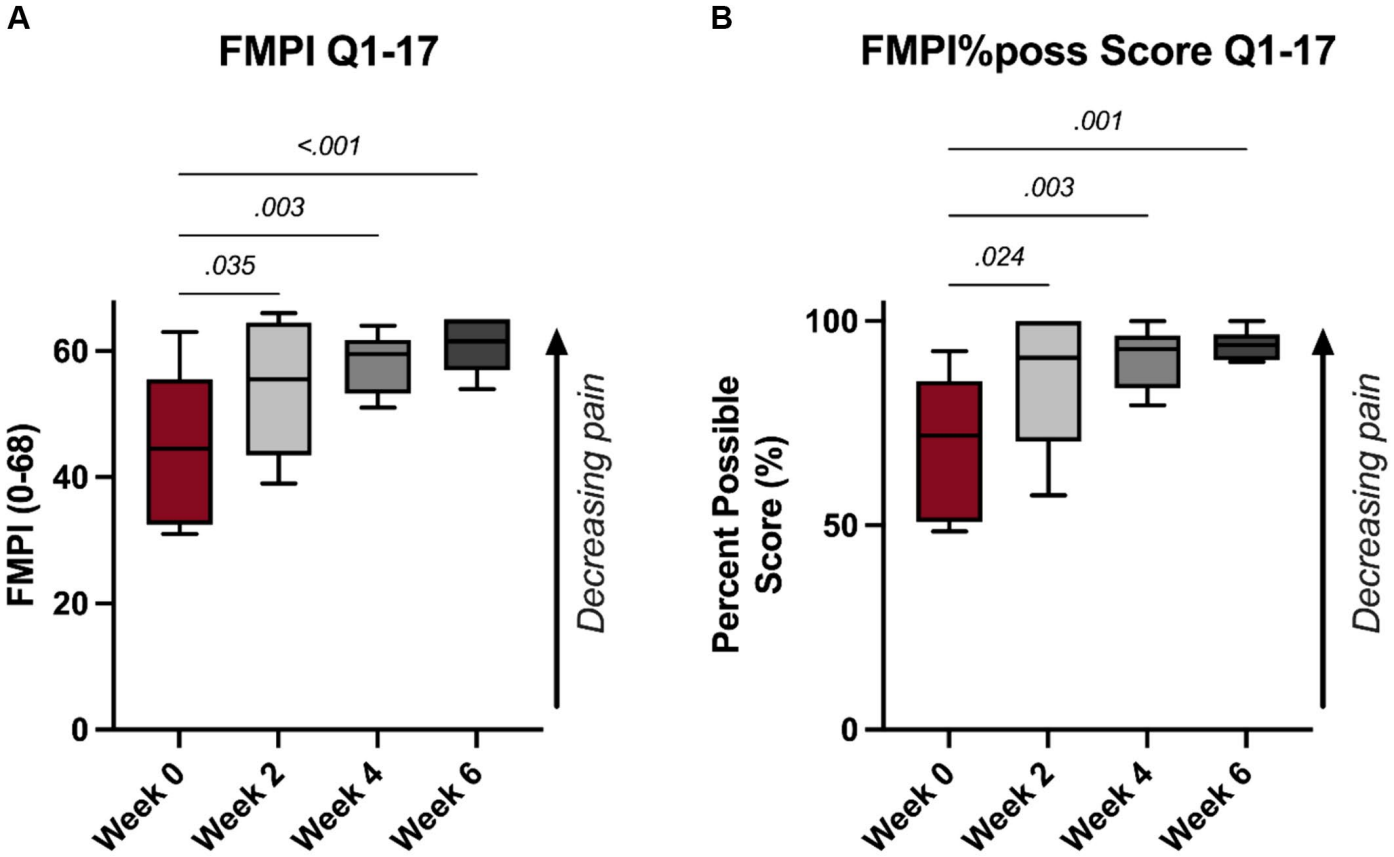
**(A)** FMPI questions 1–17 scoring and **(B)** FMPI questions 1–17%possible score.

A significant improvement in FMPI scores were noted following PRP injections. Specifically, significant improvements were noted as early as 2-week (*p* = 0.035, Figure 1A), with monotonic increases in FMPI scoring through the 6-week timepoint, at which the mean FMPI Q1-17 score improved to 60.8 ± 4.4 from 44.8 ± 12.2 at 0-week (*p* < 0.001, Figure 1A). FMPI%poss Q1-17 scoring illustrated similar improvements following PRP injections, with a similar monotonic increase in normalized FMPI scores persisting through the 6-week timepoint (*p* ≥ 0.024, Figure 1B). All FMPI and VAS scoring results for each patient are detailed in Table 1.

**Table 1:** FMPI, FMPI%poss, and VAS scoring for each patient.

	FMPI						FMPI%poss score						Clinician VAS Scoring			
	Q1–17				Q1–19		Q1–17				Q1–19					
Patient ID	Week 0	Week 2	Week 4	Week 6	Week 0	Week 6	Week 0	Week 2	Week 4	Week 6	Week 0	Week 6	Week 0	Week 2	Week 4	Week 6
1	31	45	51	54	-	62	51.7	75.0	85.0	90.0	-	90.5	40	80	80	80
2	42	60	60	60	48	68	65.6	100.0	100.0	100.0	66.0	100.5	40	60	80	80
3	33	39	54	63	36	69	48.5	57.4	79.4	92.7	48.7	93.0	70	80	100	90
4	47	51	59	65	51	72	78.3	85.0	92.2	95.6	78.6	96.0	40	15	50	90
5	53	64	61	58	57	66	82.8	100.0	95.3	90.6	83.1	91.1	30	95	90	90
6	63	66	64	65	69	73	92.7	97.1	94.1	95.6	93.0	96.1	80	90	90	95

**Figure 2:**
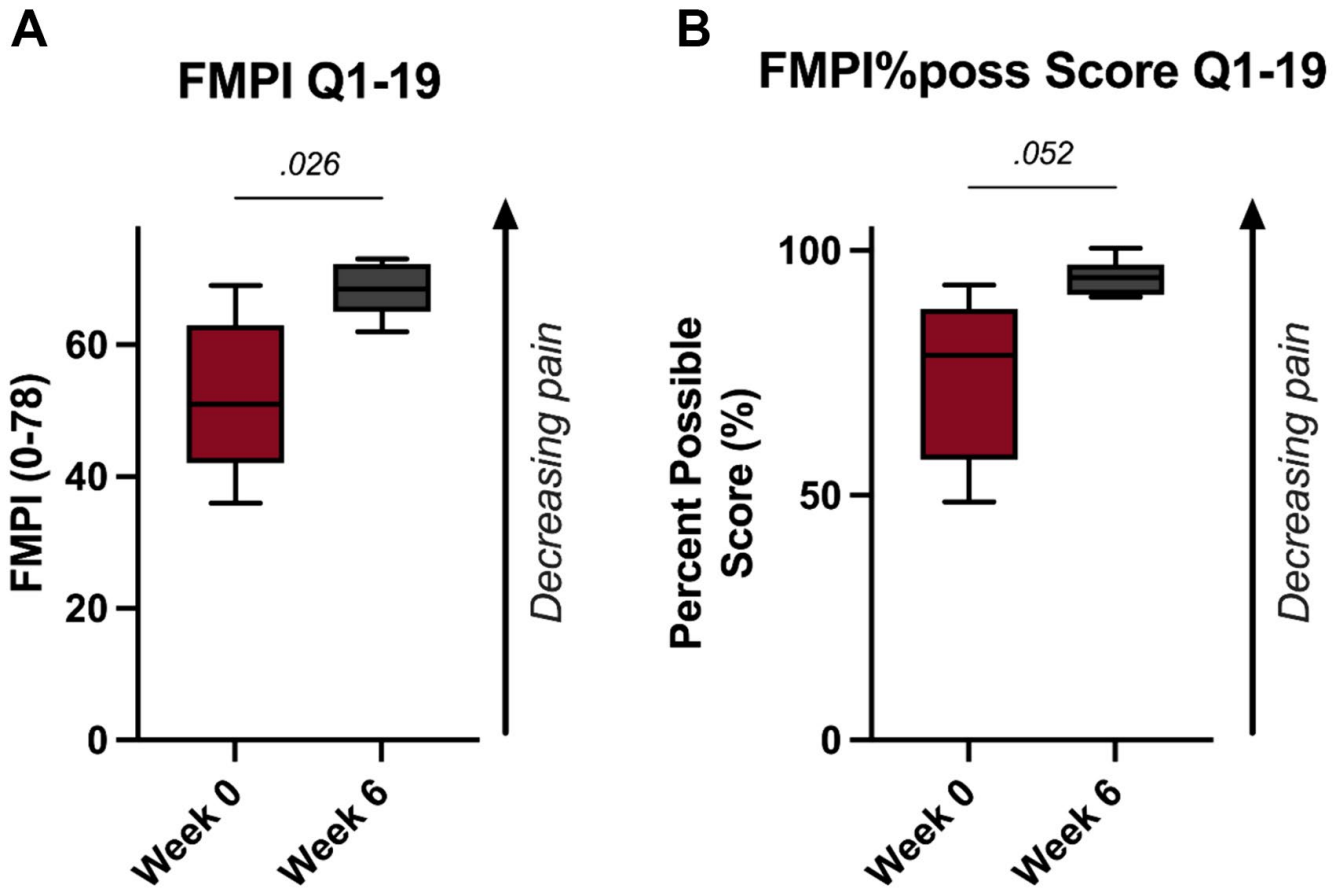
**(A)** FMPI questions 1–19 scoring and **(B)** FMPI questions 1–19%possible score.

These improvements were substantiated with the FMPI Q1-19 scoring. Specifically, scoring revealed a significant improvement in feline pain by the 6-week timepoint as measured by the FMPI Q1-19 and FMPI%poss Q1–19 scoring (*p* = 0.026, Figure 2A; *p* = 0.052, Figure 2B).

### Clinician VAS scoring results

**Figure 3:**
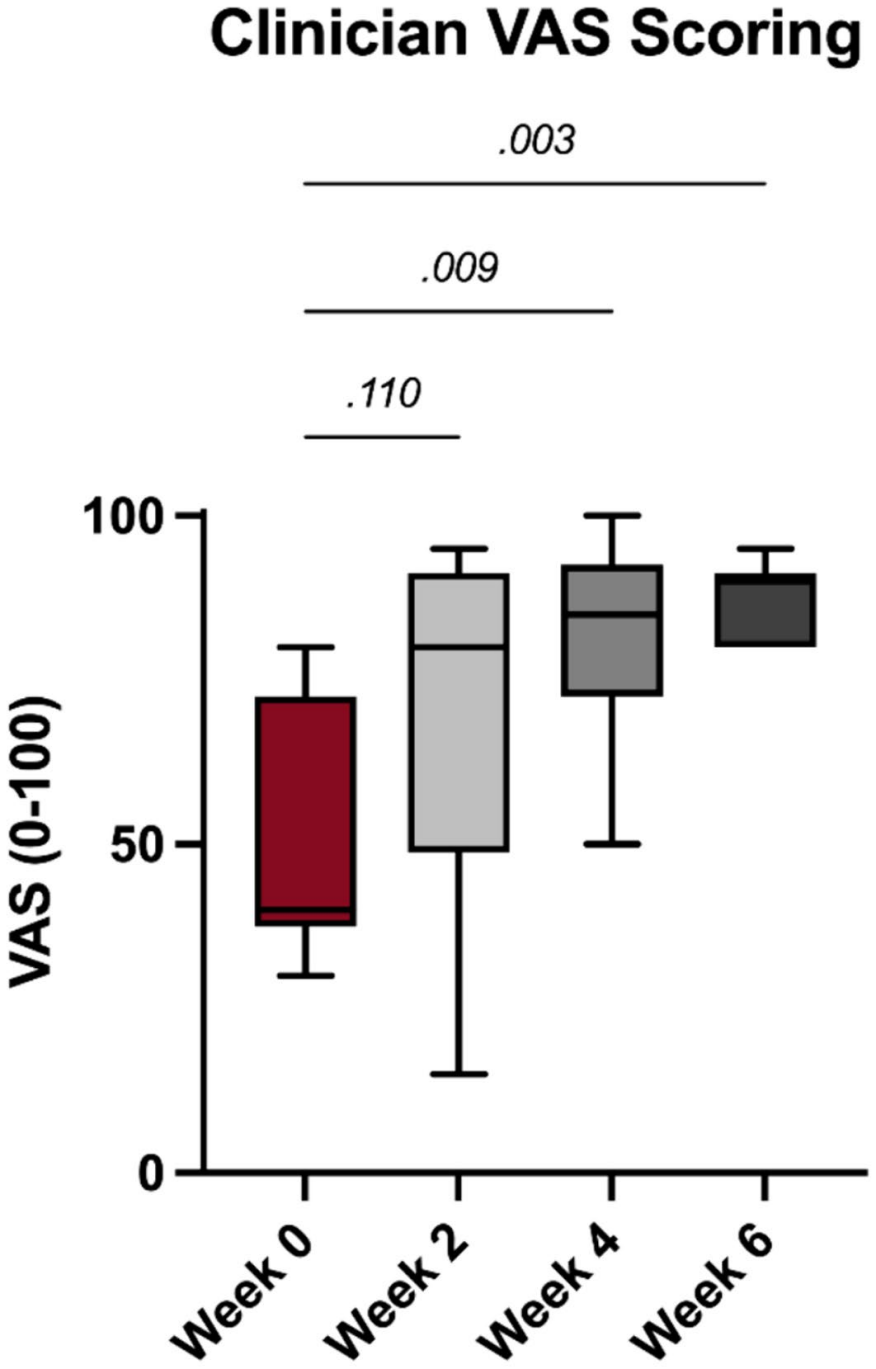
Clinician visual analog scale (VAS) results.

Significant improvements in clinician noted feline pain following PRP injections were noted at the 4- and 6-week timepoints (*p* = 0.009 and 0.003, Figure 3).

### Adverse events

No adverse events were noted throughout the course of this study.

## Discussion

These pilot data demonstrated a significant decrease in feline pain and disability over a short 6-week timespan following IA administration of autologous PRP injections for DJD/OA. Degenerative joint disease is a common cause of pain and discomfort in older cats, in some studies reported to affect up to 90% of the patient population (26). This condition impacts both feline quality of life and bond with pet owner (27). These data suggest that autologous PRP administration as prepared with the CRT system can provide an effect means of treating feline DJD/OA.

While this is the first study to report the use of autologous PRP in treatment of feline DJD, positive effects have been previously reported in other species. Several publications have reported a positive effect of IA PRP in the management of canine OA, both in surgically induced models and with naturally occurring OA (28–31). These studies in dogs with OA have demonstrated improvements in various outcome measures, including validated client surveys and subjective pain/gait scores at timepoints ranging from 30 to 180 days, with both single PRP injections as well as a regimen of a 2 IA injection series 14 days apart (28, 29, 32, 33). Taken together, leading evidence suggests IA administration of autologous PRP can effectively provide improvements in mobility and decreases in pain in these companion animals afflicted with DJD/OA.

Multiple preparations of PRP have been developed and studied in humans, canines, felines, and equines; however, the constitutional makeup of the PRP varies greatly depending on the manufacturer, species, and method of processing, making equipment/processing specific validation important (19, 20, 22, 34). Additionally, the constituent concentrations needed for a product to be considered PRP and have clinical efficacy are debated, with suggestions of platelet concentrations ranging from 1.5 to 5x those seen in whole blood (35–40). While there is debate on whether various other constituents should be included or removed, recent studies have found that red blood cells (RBCs), neutrophils, and mononuclear cells may also affect the efficacy of a PRP product and how it contributes to the inflammatory response following PRP administration (19, 41). RBCs and neutrophils are believed to be detrimental to the efficacy of intra-articular PRP as they induce unwanted inflammatory mediators that can cause synoviocyte death and impede intra-articular healing (41, 42). Two previous published studies have quantitated key parameters of the PRP product produced from healthy adult felines by the same commercially available PRP system used in this study (22, 34). Both studies showed a 151–187% increase in platelet concentration and significantly reduced RBC and neutrophil concentration from baseline WB on average. The data presented herein suggest that feline autologous PRP prepared using the CRT system effectively concentrates the platelet concentration to therapeutic levels, as demonstrated by the significant improvements in FMPI and clinician VAS scores.

Feline platelets are larger than those of other species, with a mean volume of 11.0–18.1 fL (43), compared with platelet volumes in the dog, pig and human of 7.6–8.3 fL and in the ox, horse, sheep, rat, guinea pig and mouse of 3.2–5.4 fL (44). Larger platelets have a greater number of granules, greater capacity for protein synthesis, greater amounts of secretory products compared to smaller platelets and hence are thought to be functionally more active (45, 46). Additionally, certain features of feline platelets may result in them being more reactive than those of other species: higher concentration of interactive peptides including serotonin (45) and, uniquely among domesticated species, response to serotonin by irreversible platelet aggregation with granule release (47).

The alpha granules are thought to be a rich source of growth factors as their constituents regulate angiogenetic pathways and stimulate the natural healing cascade to govern repair mechanisms to reconstruct tissue structures and ultimately restore structure and function. But the modulatory and intercellular communicative nature of the platelets may be more due to the extra-granular cytoskeletal structure than alpha granular content (48). Cat platelets once activated, have a unique microtubular and filamentous web that surrounds and protects the granules, and is responsible for the light blue appearance of the platelet cytoplasm, often mistaken for a nucleus. This is also responsible for the variability in size of the platelets and thought to be attributed to the M loop region of the alpha-1 tubulin gene within the feline genome (49).

The microtubules and cytoplasmic filaments of platelets are thought to be extremely important in granule trafficking and release, but are ultimately responsible for the formation of fibrin, healing cartilage, and commencing collagen production (50). The microtubule-binding proteins, dynein, dynactin, and kinesins, which contribute to the so called “motor activity” along the tubules, also provides structural support for the platelets, particularly when they are subjected to shearing forces in both circulation and in high friction wound injury (51), making platelets uniquely suited to high motion environments such as joints.

The microfilament and tubulin environment is so important that in 2009, Ehrenfest introduced re-categorizing platelet-rich preparations based on two factors: the presence of leukocytes and the density of the fibrin network. Current thought process has pivoted away from the optimum alpha granular content and focused more on platelet, macrophage, and monocyte function, so called “platelet dose”; high platelet doses in clinical PRP preparations are essential to implementing orthobiological treatment strategies, because platelet extragranular meshing, and leukocytic constituents interact intensely with angiogenic activities, stimulating (neo) angiogenesis to re-establish the microvascular architecture in pathological tissue structures (48). In this study, we hypothesized that the factors present in feline platelet preparations, especially those concentrated using the CRT system employed here, could decrease synovial inflammation and enhance cartilage healing when administered intra-articularly in this species.

While there are medications that may be used in the management of chronic pain in cats (e.g., Monteiro), many are lacking scientific data on their safety or efficacy for long-term treatment. The recent addition of anti-NGF antibody injections to the armament for treatment of OA pain in cats has been welcomed but sadly provides limited duration of relief (1 month) Additionally, orally administered treatments in felines may be challenging from a palatability and ease of administration standpoint. For these reasons and considering the data presented herein, IA PRP injections may be a suitable longer term therapeutic replacement for pharmaceutical pain management in this patient population.

The primary limitations of this work stem from the small samples size and relatively limited outcome measures. Determination of PRP constituents would have enabled correlation between therapy constituents and patient outcomes; however, this was not deemed practical in this pilot study. Of note, previous work has reported constituent concentrations using this same commercial kit in a separate feline study (22, 34). However, previous research has demonstrated that the discriminatory validity and sufficient repeatability of the FMPI scoring (24, 25), suggesting the outcome measures utilized herein are appropriate and representative of true improvements in feline DJD/OA-related pain/disability. Additionally, the clinician- and owner-reported results presented herein report paralleled timing and magnitude of pain reduction, supporting claim validity. However, a caregiver placebo effect in owner reports during the treatment period cannot be discounted. While owner expectation of a positive effect and decreased awareness of owners to their cat’s behaviors cannot be discounted, this study mitigated that risk through owner training/counseling on recognition of signs of pain in their cats, as previously recommended (23). To adjust for initial caregiver placebo effect, future research might use a crossover study design. Future work should entail larger studies that include longer follow up periods to elucidate the temporal benefits of IA PRP administration, as well as the potential for further improvements following multiple administrations. Additionally, comparisons assessing the impact of similar therapies (e.g., autologous conditioned serum and other PRP formulations) should be conducted to determine optimum constituent(s).

This pilot study demonstrates the potential of IA autologous PRP as prepared with the CRT system in improving clinical outcomes for cats suffering from DJD. The significant improvements in both owner-perceived FMPI scores and clinician-assessed VAS scores at the 6-week mark highlight PRP’s efficacy in enhancing joint function and reducing pain. These promising results suggest that PRP therapy could be a valuable addition to the current treatment modalities for feline DJD. However, further research with larger sample sizes and longer follow-up periods is essential to fully ascertain the long-term benefits and optimal treatment protocols of PRP in feline orthopedic conditions.

## Data availability statement

The raw data supporting the conclusions of this article will be made available by the authors, without undue reservation.

## Ethics statement

The animal studies were approved by Ethics Committee Essex Animal Hospital and Central Hospital for Veterinary Medicine. The studies were conducted in accordance with the local legislation and institutional requirements. Written informed consent was obtained from the owners for the participation of their animals in this study.

## Author contributions

JH: Conceptualization, Data curation, Funding acquisition, Investigation, Methodology, Resources, Writing – original draft, Writing – review & editing. AL: Data curation, Writing – original draft, Writing – review & editing, Conceptualization, Investigation, Methodology, Project administration, Resources, Supervision, Validation. JJ: Data curation, Writing – original draft, Writing – review & editing, Formal analysis, Visualization. LM: Conceptualization, Data curation, Formal analysis, Funding acquisition, Investigation, Methodology, Project administration, Resources, Software, Supervision, Validation, Visualization, Writing – original draft, Writing – review & editing.
